# Removal Effect of Basic Oxygen Furnace Slag Porous Asphalt Concrete on Copper and Zinc in Road Runoff

**DOI:** 10.3390/ma14185327

**Published:** 2021-09-16

**Authors:** Tianyuan Yang, Meizhu Chen, Shaopeng Wu

**Affiliations:** State Key Laboratory of Silicate Materials for Architectures, Wuhan University of Technology, Wuhan 430070, China; yangty2017@whut.edu.cn (T.Y.); wusp@whut.edu.cn (S.W.)

**Keywords:** heavy metals, road runoff, porous asphalt pavement, steel slag, removal effect

## Abstract

In order to improve the utilization efficiency of road runoff and the remove effects of heavy metals, porous asphalt pavements have been used as an effective measure to deal with heavy metals in road runoff. However, the removal effect on dissolved heavy metal is weak. In this paper, basic oxygen furnace (BOF) slag was used as aggregate in porous asphalt concrete to improve the removal capacity of heavy metal. Road runoff solution with a copper concentration of 0.533 mg/L and a zinc concentration of 0.865 mg/L was artificially synthesized. The removal effect of BOF slag porous asphalt concrete on cooper and zinc in runoff was evaluated by removal tests. The influence of rainfall intensity and time on the removal effect was discussed. The results obtained indicated that BOF slag porous asphalt concrete has a better removal effect on copper. The removal rate of copper is 57–79% at the rainfall intensity of 5–40 mm/h. The removal rate of zinc is more susceptible to the changes of rainfall intensity than copper. The removal rate of zinc in heavy rain conditions (40 mm/h) is only 25%. But in light rain conditions (5 mm/h), BOF slag porous asphalt concrete maintains favorable removal rates of both copper and zinc, which are more than 60%. The heavy metal content of runoff infiltrating through the BOF slag porous asphalt concrete meets the requirements for irrigation water and wastewater discharge. The results of this study provide evidence for the environmentally friendly reuse of BOF slag as a road material and the improvement of the removal of heavy metal by porous asphalt concrete.

## 1. Introduction

With the rapid development of urbanization and the continuous increase of highway mileage and traffic volume, soil pollution and water pollution caused by highways have gradually attracted attention. Various pollutants from vehicles or the atmosphere accumulate on the road during non-rainfall periods, and when it rains, the accumulated road pollutants are easily washed by rainfall runoff and carried into the groundwater or soil, causing potential harm to the surrounding environment [1,2]. In addition to conventional pollutants such as suspended solids and organic pollutants, road runoff also contains a variety of heavy metals, which are difficult to degrade in the environment, and the harm to organisms is generally irreversible for life [3,4]. Research has shown that only 40–50% of pavement pollutants can be removed by conventional cleaning measures [5]. Mechanical cleaning can only remove particles above 250 microns in size, and has little effect on dissolved heavy metal [6]. 

In order to remove road runoff pollutants more efficiently, advanced measures have been applied to road engineering such as vegetation control, wet retention ponds, and infiltration systems [7]. The vegetative buffer strips reduced the runoff volume by 35–90%, sediment concentration by 42–94%, nitrate concentration by 35–88% and phosphate concentration by 28–95% [8]. Wet retention ponds can reduce the dissolved nitrogen species, total and dissolved phosphorus, and total suspended solids concentrations for more than 30% [9]. Porous asphalt pavement is a type of road infiltration system. In addition to improving the anti-slide performance of the pavement and reducing traffic noise, the purpose of early research and application of porous asphalt pavement is to reclaim stormwater and recharge the groundwater. As researchers continued to study porous asphalt pavements, it was realized that the porous asphalt pavement has a good control effect on the suspended solids and heavy metal in the road runoff due to the complex pore structure [10,11,12,13]. In a laboratory-scale test, porous asphalt concrete prepared with limestone and basalt produced average reductions in zinc of 79% during the 696 h storage [14]. But, the removal rate was lower (less than 40%) at the initial stage (0–144 h). It is indicated that basalt and limestone have a removal capacity on zinc indeed, but it takes a long time to take effect. The removal mechanism of heavy metal ions in pavement runoff by porous asphalt pavement includes adsorption, precipitation and electrostatic attraction [15,16,17]. Precipitation and electrostatic attraction generally originate from the interaction between heavy metal ions and aggregates of porous asphalt pavement. For instance, the surface components of calcite can be complexed with Zn^2+^ and Pb^2+^ to form carbonate precipitates [15]. The negative charge on the surface of dolomite has electrostatic adsorption of heavy metal cations [17].

Basic oxygen furnace (BOF) slag is a kind of steel slag. As a by-product in the steelmaking process, steel slag is often recycled for road construction due to its good mechanical properties [18,19]. Steel slag with a particle size of 2.36–25 mm is usually used as an aggregate for pavement engineering, which can enhance the ability of the pavement to resist heavy loads, so that steel slag improves the durability of the asphalt pavement [20]. Moreover, steel slag significantly improves the anti-skid performance, bending strength and Marshall stability of asphalt concrete [21,22]. X-ray fluorescence spectroscopy results show that steel slag generally contains 25–50% calcium oxide, 25–40% iron oxide (or ferric oxide), 8–18% silicon dioxide, 1–9% aluminum oxide, 3–13% oxidase, and other components [20]. Calcium oxide is one of the main components of steel slag. Most of the calcium oxide combines with silicon dioxide to form calcium silicate, the others are free calcium oxide (f-CaO). f-CaO will undergo a hydrolysis reaction in the aqueous solution to release hydroxyl ion, which results in the alkalinity of the aqueous solution [23]. Therefore, steel slag has a chemical precipitation and acid neutralizing capacity. Studies have shown that steel slag can effectively remove chromium, copper, zinc, lead and other metals in wastewater [24,25,26,27]. In the cylindrical column test, the removal efficiency of steel slag for zinc and copper were 78% and 68% [26]. Otherwise, steel slag has a maximum removal efficiency of 74%, 64% and 34% for chromium, cadmium and cadmium, respectively [27]. Recycling steel slag as aggregates can not only reduce the consumption of natural resources, but also theoretically improve the removal of heavy metal by porous asphalt concrete. However, when steel slag is used as aggregate, its surface is wrapped by asphalt, and the leaching of free oxides is inhibited. The generation of hydroxide ions is reduced, resulting in a weakening of the sedimentation effect on metal cations. There are few relevant studies at present, so the actual effect of steel slag porous asphalt concrete in purifying road runoff remains to be explored.

Although there are a variety of methods to deal with pollutants in road runoff, only a few have obvious effects on heavy metals, and include the disadvantage of low efficiency. Using steel slag as the aggregate of asphalt concrete to precipitate heavy metals by increasing the pH of the solution is a more efficient way to take effect. As steel slag is a kind of solid waste, the reuse of resources is realized at the same time. In this study, steel slag and basalt were used as aggregates to prepare porous asphalt concrete, and their volume properties and road performance were characterized. According to the observation data of road rainwater runoff in the literature, a rainwater runoff solution containing Cu^2+^ and Zn^2+^ was artificially synthesized. The removal rate of copper and zinc by porous asphalt concrete under different conditions was measured by removal tests. The effects of steel slag porous asphalt concrete on the removal of copper and zinc in road runoff and the influence of rainfall intensity and duration were evaluated. The results of this study provide evidence for the reuse of steel slag as a road material and the improvement of the removal of heavy metal by porous asphalt concrete.

## 2. Materials and Methods

### 2.1. Raw Materials

In this study, BOF slag and basalt from Hubei Province were used as the aggregates. BOF slag generally contains a higher content of calcium oxide than other steel slags, which results in a higher alkalinity. The chemical composition of BOF slag and basalt was obtained by X-ray fluorescence analysis (XRF), as shown in Table 1. Most of the calcium oxide in the steel slag combines with silicon dioxide to form calcium silicate. When the ratio of calcium oxide to silicon dioxide is high, free calcium oxide exists. The BOF slag used in this paper has a ratio of calcium oxide to silica content exceeding 2.5, which is a steel slag with high basicity [20]. The ratio of calcium oxide to silicon dioxide in basalt is so low that there is little free calcium oxide. The main properties of BOF slag and basalt are shown in Table 2, which meet the requirements in current Chinese standard [28]. Limestone powder with a size less than 0.075 mm was used as a filler of asphalt concrete.

In order to prepare porous asphalt concrete with large porosity, modified asphalt with higher viscosity is required to provide sufficient cohesive strength. SBS modified asphalt has better viscosity and high temperature stability than base asphalt. In this study, SBS modified asphalt with a penetration of 46.8 (0.1 mm at 25 °C, 100 g, and 5 s), ductility of 45.0 cm (5 cm/min, 5 °C), and softening point of 78.3 °C was used as the binder. 0.05 mol/L Zn(NO_3_)_2_ solution, 0.05 mol/L Cu(NO_3_)_2_ solution and deionized water were used to artificially synthesize road runoff solution because nitrate ions would not interfere with the removal tests.

### 2.2. Experimental Preparation

#### 2.2.1. Porous Asphalt Concrete Specimens

**Figure 1:**
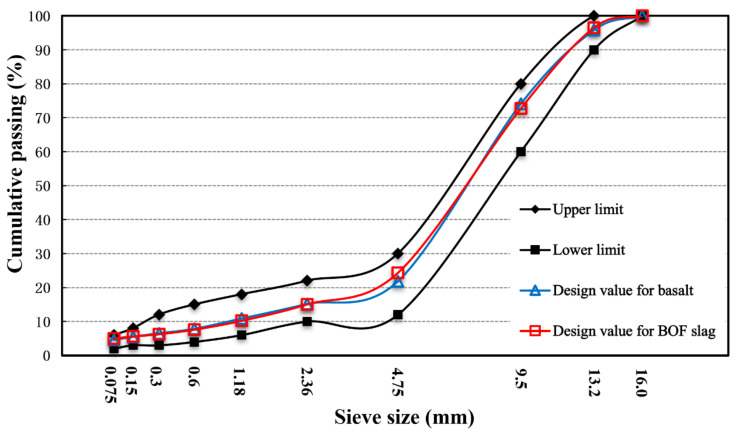
Gradation curves of basalt and BOF slag.

The aggregate gradation of OGFC-13 was used in the paper, and the optimal asphalt content was determined by a series of tests for porous asphalt concrete [29]. The design results of the aggregate gradation are shown in Figure 1.

The preparation of porous asphalt concrete specimens was based on the current Chinese standard [30]. According to the design results, the aggregate, filler and asphalt were mixed at 175 °C to prepare a loose asphalt mixture. The loose asphalt mixture was compacted into Marshall specimens by an electric compactor. The Marshall specimen is a cylindrical asphalt concrete specimen with a height of 63 mm and a diameter of 101 mm. The basic properties of Marshall specimens, including Marshall stability, immersion residual stability and freeze-thaw residual strength, were specified in the current Chinese standards [30,31]. The void ratio of BOF slag Marshall specimens and basalt Marshall specimens were 22.4% and 21.6%, respectively.

In order to increase the length-diameter ratio to ensure uniform infiltration, three Marshall specimens were stacked as the porous asphalt concrete specimens in each removal test. The height of three Marshall specimens was about 189 mm, which is close to the thickness of the asphalt pavement surface. The removal tests were also applied to the loose asphalt mixture as a comparison.

#### 2.2.2. Synthetic Stormwater Runoff

Zhang [32] summarized the concentrations of heavy metal pollutants lead, zinc, and copper in road runoff in worldwide research. Among them, the concentration of zinc is generally the highest, and the concentration of lead and copper are close to each other, but the concentration of copper has a higher peak. In addition, studies have pointed out that copper in runoff exists in both dissolved and granular states and can be transformed into each other, while lead mostly exists in granular states [33,34,35]. The selection of BOF slag as aggregate in this paper mainly improves the sedimentation effect of porous asphalt concrete on dissolved heavy metal. Therefore, the rainwater runoff solution was synthesized according to the concentration range of copper and zinc in the literature, and the concentration is shown in Table 3.

### 2.3. Experiments and Procedures

**Figure 2:**
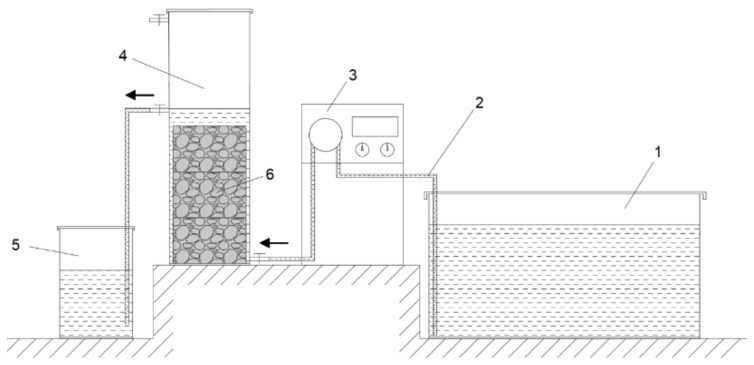
Laboratory scale road runoff heavy metal removal experimental facility. 1—liquid storage vessel, 2—rubber tube, 3—peristaltic pump, 4—reaction vessel, 5—collection vessel, 6—asphalt concrete specimens.

In this paper, removal tests were undertaken to allow the synthetic runoff solution infiltrating through the porous asphalt concrete or loose asphalt mixture as a contrast. In the process, calcium hydroxide was produced by the hydration of f-CaO, which made the solution alkaline. The Cu^2+^ and Zn^2+^ in the solution were precipitated by OH^−^ and adsorbed by the complex voids in the porous concrete. In order to achieve the above experimental process, an experimental facility as shown in Figure 2 was designed.

The experimental device was mainly composed of four parts: liquid storage vessel, peristaltic pump, reaction vessel and collection vessel, which were connected by rubber tubes. The liquid storage vessel was used to store the synthetic runoff solution, and the peristaltic pump could deliver the solution to the reaction vessel at a certain flow rate. The reaction vessel was cylindrical, in which the asphalt concrete specimens or loose asphalt mixture was placed. The diameter of the reaction vessel was 105 mm. There was a liquid inlet at the bottom of the side wall of reaction vessel, and a liquid outlet at the heights of 200 mm and 300 mm, respectively, on the opposite side. The liquid outlet at 200 mm was for the stacked Marshall specimens, and 300 mm was for the loose mixture, because the loose mix of the same quality is higher than the compacted Marshall specimens. The runoff solution was from bottom to top in the vessel to make the solution infiltrate evenly through the concrete specimens under the action of gravity. Three Marshall specimens were stacked in the reaction vessel to simulate a porous asphalt concrete pavement. The peristaltic pump was set to the flow rate required for the experiment. Since the diameter of the reaction vessel was 105 mm, a flow rate of 1.44 mL/min was used to simulate a rainfall of 10 mm/h, according to the calculation formula of the cylinder volume. Similarly, 0.72 mL/min, 2.88 mL/min, 4.33 mL/min and 5.77 mL/min were used to simulate 5 mm/h, 20 mm/h, 30 mm/h and 40 mm/h rainfall, respectively. The effluent runoff solution flowed into the collection vessel through the rubber tube.

The runoff solution in the collection vessel was stirred well every 8 h and cleaned up after sampling. A pH meter was used to measure the pH value of each liquid sample. Inductively coupled plasma atomic emission spectrometry was used to analyze the heavy metal composition and concentration in the liquid samples. The composition of liquid samples under different conditions was compared, and the effect of BOF slag porous asphalt concrete on the removal of heavy metal in road runoff and its influencing factors were evaluated.

## 3. Results and Discussion

### 3.1. Removal Effect of Basic Oxygen Furnace (BOF) Slag Asphalt Concrete on Copper and Zinc

**Figure 3:**
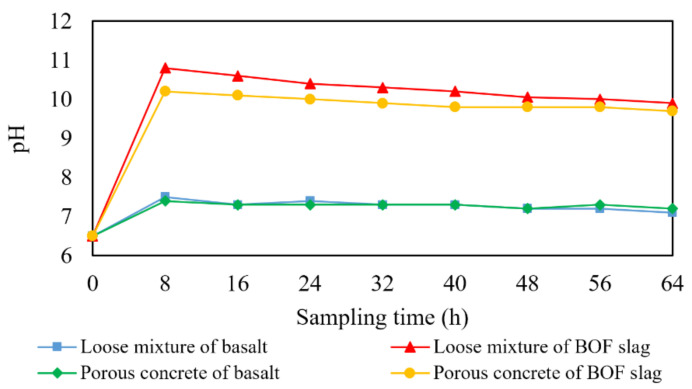
pH vs. time of runoff solution infiltrating through different specimens.

Removal tests were carried out on BOF slag porous asphalt concrete, basalt asphalt concrete and their loose asphalt mixtures. The solution flow rate was set to 1.44 mL/min to simulate a rainfall intensity of 10 mm/h. During the process, the pH changes of the effluent runoff solution were recorded, as shown in Figure 3. 

The value of 0 h represents the pH of the solution before tests. The synthetic runoff solution is composed of Cu(NO_3_)_2_ and Zn(NO_3_)_2_, which are hydrolyzed to make the solution weakly acidic. When the solution infiltrates through the BOF slag porous asphalt concrete, the f-CaO in the BOF slag hydrates and the solution becomes alkaline. The BOF slag with the size of 5–10 mm can make the pH of the solution up to 11–12.5 [36]. But the pH of the solution in this paper is only 10–11, because the BOF slag used as aggregate was mainly 4.75–16 mm in size and coated with asphalt. The pH of the solution infiltrating through the loose mixture of BOF slag was higher than the porous asphalt concrete and decreased faster because of larger contact area between BOF slag and solution, which indicates that the skeleton structure of asphalt concrete inhibited the hydration of f-CaO in BOF slag and slowed down its loss. There was little free alkaline oxide in basalt, so the basalt asphalt mixture had little effect on pH.

**Figure 4:**
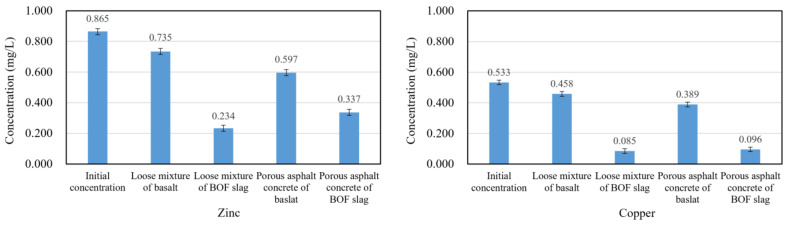
Concentration of zinc and copper in runoff solution at 8 h.

**Figure 5:**
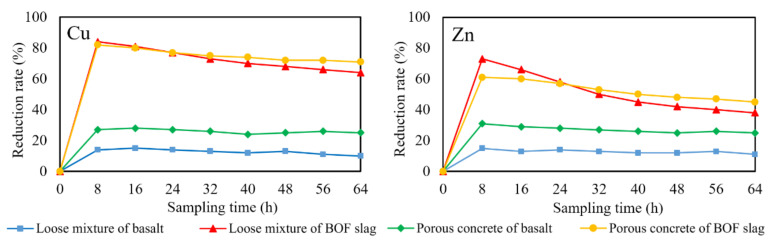
Reduction rate of copper and zinc in runoff solution infiltration through different specimens.

The effluent runoff solution samples were collected every 8 h, and the composition was analyzed by inductively coupled plasma emission spectrometer. All the results were the average of three measurements. The error was less than 10%. Concentration of copper and zinc in runoff solution at 8 h are shown in Figure 4. The concentration of zinc and copper in the liquid samples infiltrating through BOF slag specimens were much lower than basalt specimens, which indicated that BOF slag can improve the effect of porous asphalt concrete on the removal of zinc and copper. The removal rate of copper and zinc by different specimens was calculated according to the concentration difference of copper and zinc before and after tests, as shown in Figure 5.

The removal rate of both copper and zinc by BOF slag is higher than that of basalt. However, the reduction rate curves of basalt are relatively flat, indicating that the pore structure in the asphalt mixture has a stable removal effect on heavy metal. The removal capacity of porous asphalt concrete is higher than that of loose mixture. However, the removal effect of the loose asphalt mixture of BOF slag on copper and zinc is better than BOF slag porous asphalt concrete in the initial stage. This is because the f-CaO in the loose mixture is easier to hydrate in order to provide a higher pH of the solution, as shown in Figure 3. It is indicated that the sedimentation effect of the alkaline oxides in the BOF slag on heavy metal is much stronger than the removal effect of the pore structure in the initial stage. However, the content of f-CaO in BOF slag is limited, so the sedimentation effect gradually weakens with the leaching of f-CaO, resulting in a rapid drop in the reduction rate of heavy metal, which is more obvious on the curve of zinc. Although it is hard to dissolve both zinc hydroxide and copper hydroxide in water, the solubility product of zinc hydroxide is larger, so the removal rate of zinc is more susceptible to the changes in pH. The removal rate of zinc by loose asphalt mixture of BOF slag was reduced to about 40% at 64 h, while the curve of BOF slag porous asphalt concrete was flatter. It was demonstrated that the removal effect of pore structure of BOF slag porous asphalt concrete plays an important role in the removal of zinc.

### 3.2. Influence of Rainfall Intensity on the Reduction Rate of Heavy Metal

**Figure 6:**
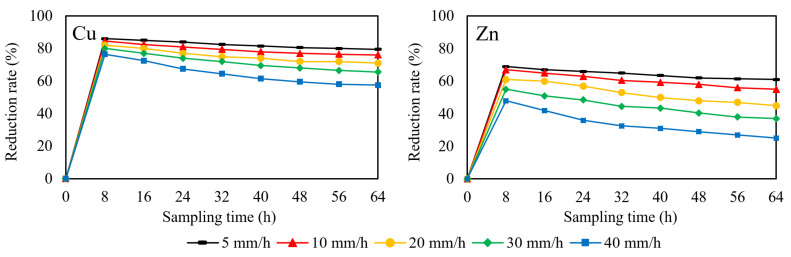
Reduction rate of copper and zinc at different rainfall intensity.

In order to investigate the influence of rainfall intensity on the reduction rate of heavy metal, the flow rate was set to 0.72 mL/min, 2.88 mL/min, 4.33 mL/min and 5.77 mL/min to simulate rainfall intensity of 5 mm/h, 20 mm/h, 30 mm/h and 40 mm/h, respectively. The specimens in this section was BOF slag porous asphalt concrete only. The results of removal tests are shown in Figure 6. 

The reduction rate of copper and zinc is decreased by the increase of rainfall intensity and sampling time. The decrease of zinc reduction rate is particularly obvious. The increase in rainfall intensity means that more runoff infiltrate through the concrete in the same period of time, resulting in a faster decrease in pH. The steeper curve of zinc also confirms the inference that the removal of zinc is more susceptible to pH changes. However, BOF slag porous asphalt concrete still maintains a good removal effect on zinc at a rainfall intensity of 5 mm/h, which is over 55%. The removal rate of copper is relatively steady, which is more than 70% at the rainfall intensity of 20 mm/h. Rainfall intensity higher than 20 mm/h is rare, so that BOF slag porous asphalt concrete has a good effect on removing copper in most rainfall situations, and effectively removes zinc under light rain conditions. Compared with the results in the literature, the porous asphalt concrete prepared with limestone and basalt produces an obvious effect (more than 40%) on the removal of zinc only after 168 h storage with the initial concentration of 0.51 mg/L, while the BOF slag porous asphalt concrete provides a stabilizing effect (45–70%) for the removal of zinc from the beginning with the similar initial concentration (0.865 mg/L). It is indicated that BOF slag, as the aggregate of porous asphalt concrete, provides an efficient removal effect on zinc and copper. The final concentrations of copper and zinc are 0.096–0.227mg/L and 0.337–0.649 mg/L, respectively, under different rainfall conditions, which meet the Chinese standards for wastewater discharge and irrigation water [37,38].

### 3.3. Additional Heavy Metal Brought by BOF Slag Aggregate

In addition to zinc and copper, multiple heavy metals exist in BOF slag aggregate, which may leach into the runoff. The leaching concentration of heavy metals in steel slag asphalt mixture shows a downward trend with time [18]. Therefore, the liquid sample of the early stage (8 h) at 10 mm/h rainfall intensity was chosen to analyze the concentration of heavy metal, as shown in Table 4. The results were compared with current Chinese standards [37,38,39]. The concentration of heavy metal including copper and zinc meets the requirements for wastewater discharge and irrigation water. The concentrations of manganese, chromium and lead meet the requirement for groundwater (Class I). It is indicated that BOF slag porous asphalt concrete removes the original heavy metal in the runoff while it produces little additional heavy metal.

## 4. Conclusions

In this paper, the removal effect of BOF slag porous asphalt concrete on copper and zinc in runoff was evaluated by removal tests, and its influencing factors were explored. On the basis of the data obtained in this study, the following conclusions are drawn:

(1) The BOF slag porous asphalt concrete can turn the pH of the runoff solution up to 10–11, which significantly increases the removal rate of copper. The pore structure of porous asphalt concrete plays an important role in the removal of zinc.
(2) With the increase of rainfall intensity and time, the removal rate of copper and zinc gradually decreases. The removal rate of zinc is more susceptible to the changes of rainfall intensity than copper. However, in light rain conditions, BOF slag porous asphalt concrete maintains favorable removal effect on both copper and zinc.
(3) While removing the original heavy metal in the runoff, the BOF slag porous asphalt concrete leaches little additional heavy metal. The heavy metal content of runoff solution infiltrating through the BOF slag porous asphalt concrete meets the requirements for irrigation water and wastewater discharge.

The results obtained indicate that BOF slag porous asphalt concrete has favorable pavement performance and significant removal effect on heavy metal in road runoff. The environmentally friendly reuse of BOF slag as a pavement material is feasible.

## Figures and Tables

**Table 1:** Chemical composition of basic oxygen furnace (BOF) slag and basalt (%).

Compound	CaO	SiO_2_	Al_2_O_3_	Fe_2_O_3_	MgO	LOI
BOF slag	45.89	18.19	1.50	23.86	6.34	0.75
Basalt	8.54	47.76	20.14	10.24	5.75	3.60

**Table 2:** Characteristics of aggregates in the asphalt mixture.

Properties		Unit	Tested Results		Requirement	Test Method
			BOF Slag	Basalt		
Apparent specific gravity	9.5–16 mm	g/cm^3^	3.634	2.960	≥2.9	T0304-2005 [28]
	4.75–9.5 mm		3.586	2.946	≥2.9	T0304-2005 [28]
	2.36–4.75 mm		3.579	2.949	≥2.9	T0304-2005 [28]
	0–2.36 mm		3.548	2.967	≥2.9	T0304-2005 [28]
Crush value		%	13.5	9.4	≤20	T0316-2005 [28]
Los Angeles abrasion		%	16.8	10.6	≤28	T0317-2005 [28]
f-CaO		%	2.7	--	≤3.0	YB/T4328-2012 [28]

**Table 3:** Target chemical makeup of synthetic stormwater runoff [32].

Item	Concentration Range in Literature	Chemical	Concentration in Synthetic Stormwater
Copper	0.01–0.85 mg/L	Cu(NO_3_)_2_ (0.05 mol/L)	0.533 mg/L
Zinc	0.03–1.76 mg/L	Zn(NO_3_)_2_ (0.05 mol/L)	0.865 mg/L

**Table 4:** Concentration of heavy metal in runoff solution infiltrating through BOF slag porous asphalt concrete.

Heavy Metals	Concentration (mg/L)		Limit Values (mg/L)		
	Initial	After Tests	Wastewater Discharge	Irrigation Water	Groundwater (Class I)
Copper	0.533	0.096	≤0.5	≤0.5	≤0.01
Zinc	0.865	0.337	≤2.0	≤2.0	≤0.05
V	0	0.032	≤1.0	--	--
Mn	0	0.004	≤2.0	--	≤0.1
Cr	0	0.003	≤0.5	0.1	≤0.01
Pb	0	0.002	≤1.0	0.2	≤0.01

## Data Availability

The raw/processed data required to reproduce these findings cannot be shared at this time as the data also forms part of an ongoing study.
